# Evaluation of the adjunctive effect of Xing Nao Jing Injection for viral encephalitis: A systematic review and meta-analysis of randomized controlled trials

**DOI:** 10.1097/MD.0000000000015181

**Published:** 2019-04-12

**Authors:** Hui-Juan Cao, Shi-Bing Liang, Wei Zhou, Jia-Rui Wu, Cheng-Liang Zhang

**Affiliations:** a Centre for Evidence-Based Chinese Medicine, Beijing University of Chinese Medicine, Beijing; b Shanxi University of Chinese Medicine, Taiyuan, Shanxi Province; c School of Chinese Pharmacy, Beijing University of Chinese Medicine, Beijing; d Department of Pharmacy, Tongji Hospital, Tongji Medical College, Huazhong University of Science and Technology, Wuhan, Hubei Province, China.

**Keywords:** meta-analysis, randomized controlled trial, systematic review, viral encephalitis, Xing Nao Jing Injection

## Abstract

**Background:**

To systematically evaluate the effect and safety of Xing Nao Jing (XNJ) injection as an add-on treatment on the treatment for viral encephalitis (VE).

**Methods:**

Trials assessing the adjunctive effectiveness of XNJ injection for VE were searched from 4 electronic databases from inception to October 31, 2018. Two authors independently extracted data and assessed risk of bias. Statistical analyses were performed using RevMan 5.3 software. Meta-analysis and additional analysis were conducted if data permitted. Trial Sequential Analysis and Grading of Recommendations Assessment, Development and Evaluation (GRADE) were also performed.

**Results:**

This review involved 23 trials and 1757 participants, all trials were assessed as having unclear risk of bias. Results from 5 meta-analyses, 13 subgroup meta-analyses, and the single studies showed that based on conventional therapy XNJ injection (0.4–0.6 mL/kg daily for children, 20 mL/day for adults) may have better effect on increasing the numbers of cured patients and decreasing the time of recovery of main symptoms for patients with viral encephalitis. Patients used combination of XNJ injection and conventional therapy had higher cured rate (risk ratio 1.61, 95% confidence interval 1.45–1.80, 19 trials, 1456 participants) and less mortality rate (risk ratio 0.26, 95% confidence interval 0.10–0.71, 9 trials, 595 participants). The average difference of time for fever, conscious, or convulsive recovery was average 2 hours shorter in combination group than in control. No difference was found between children and adults according to the subgroup analysis. Safety of the XNJ injection was failed to evaluate due to the insufficient evidence in this review.

**Conclusions:**

This review found “very low” quality evidence which showed the potential effectiveness of combination of XNJ injection and conventional therapies for VE. Considering the TSA results, conclusion could only be draw on effectiveness of the XNJ injection as add-on treatment for VE patients on increasing the cured rate. Firm conclusion on other outcome measures for effectiveness assessment or safety of XNJ injection could not be draw according to this review due to the insufficient evidence.

## 1 Introduction

Encephalitis is a disease with inflammation of the brain parenchyma, which commonly infected by viruses and presented as fever, altered level of consciousness, headache and limb paralysis.^[1]^ The estimated incidence of encephalitis has a wide variability and is dependent upon age, demographics, climate, the presence of natural host for causative agent, and the presence of epidemic illness.^[2]^ Mortality rate of acute viral encephalitis (VE) in children is 0.8% according to a China cohort study (n=261),^[3]^ another larger study^[4]^ showed the mortality rate of VE was 3.13% in 7259 patients in southeast of China.

The current treatment for VE includes antiviral therapy, immunomodulatory treatments, neuro-intensive care, and other symptomatic supportive therapies.^[5]^ However, there is still considerable sequelaes to this disorder, such as mental retardation and limb paralysis. Since consciousness is one of the main symptoms of VE, Xing Nao Jing (XNJ) Injection is commonly used for this disease in China. XNJ is extracted from a herbal patent called Angongniuhuang, the main components of it are Moschus (*She Xiang*), Borneolum Syntheticum (*Bing Pian*), Fructus Gardeniae (*Zhi Zi*), Radix Curcumae (*Yu Jin*), et al. According to TCM theory, XNJ injection has the function of clearing heat and detoxifying, cooling and invigorating the circulation of blood, as well as restoring the consciousness. Studies^[6,7]^ found XNJ may help on reducing body temperature, enhancing brain function, promoting the recovery of consciousness, and reducing the associated brain damage.

A systematic review^[8]^ with 14 included trials showed that XNJ injection plus routine therapy is superior to routine therapy alone on cure rate and symptoms decreased. However, the authors also clarified that due to the obvious clinical heterogeneity among included trials and the poor methodological quality of the included studies affect the level of the evidence. Since the previous review was published in 2013, it is worthy to update the evidence with more potential high-quality studies.

## 2 Objectives

The aim of this study is to investigate the effectiveness and safety of XNJ injection as an adjunctive therapy based on conventional treatment for viral encephalitis, and to provide the latest and rigorous evidence through evidence-based approach.

## 3 Methods

### 3.1 Criteria for considering studies for this review

Randomized controlled trials (RCTs) which compared XNJ injection with conventional therapy for patients with VE, were included in this review. VE should be diagnosed according to a recognized criterion, regardless to their age or gender. Equal conventional therapy could be used in both groups, such as antibiotics, antiviral drugs, intracranial decompression, vitamin supplement, and maintain of electrolyte balance.

The primary outcome of this review was the endpoint outcome of this disease, including the fatality rate and the cure rate. The secondary outcomes included the symptom disappearance time, the symptoms include fever, headache, vomit, convulsive, coma, et al. Adverse events was also assessed as secondary outcome. The included trials should report at least one of the above outcomes.

### 3.2 Search methods for identification of studies

PubMed, Chinese National Knowledge Infrastructure Databases (CNKI), Chongqing VIP Chinese Science and Technology Periodical Database (VIP), and Wanfang Database were searched from the inception to October 31, 2018. “Xing Nao Jing” OR “Xingnaojing” combined with “viral encephalitis” were used as subject word or MeSH word during searching, the search strategies were adjusted in different databases. Since studies concerned XNJ injection were mainly published in Chinese, only PubMed was searched for English articles which relevant.

Two authors (CHJ and LSB) screened the literatures and selected the eligible trials according to the above criteria. Disagreements were solved by discussion with the third author (WJR).

#### 3.2.1 Data collection and analysis

Two authors (CHJ and LSB) independently extracted the data and assessed the methodological quality of included trials using the risk of bias tool which recommended by the Cochrane Collaboration.^[9]^ Seven elements were assessed: random sequence generation, allocation concealment, blinding of patients, blinding of outcome assessment, incomplete outcome data (according to record the missing data and the method to deal with it), selective reporting (determined by the consistency of the predefined and reported outcomes) and other bias (assessed according to sample size calculation, inclusion/exclusion criteria for patients’ recruitment, comparability of baseline data, funding sources).

All statistical analyses were performed using RevMan 5.3 (The Cochrane Collaboration) software. Data were summarized using risk ratio (RR) with its 95% confidence interval (CI) for binary outcomes or mean difference (MD) with 95% CI for continuous outcomes. Statistical heterogeneity among included trials was measured by *I*^*2*^ statistic. Meta-analysis was conducted, if there is no obvious clinical (participants, intervention, control, and outcomes) and statistical heterogeneity (*I*^*2*^ < 75%) among included trials. When *I*^*2*^ value was <25%, we used fixed-effect model (FEM) to pool the data. When *I*^*2*^ value was between 25% and 75%, we estimated the source of heterogeneity. If the statistical heterogeneity was explained successfully by sensitive analysis or subgroup analysis (*I*^*2*^ < 25%), we also used FEM to pool the data. Otherwise, random-effects model (REM) was used. Data were not pooled when there was obvious statistical heterogeneity (*I*^*2*^>75%) unable to explain or handle (by subgroup analysis) among trials. Funnel plot was applied to explore the possibility of publication bias, when there were 10 or more trials in a meta-analysis.

Subgroup analyses were conducted to determine the evidence for different types of control (whether or not antiviral drugs used in control group) or different types of patients (children or adults) if data were available. When there were significant positive results of the outcomes, sensitive analysis was conducted to challenge the robustness of the primary analysis: trials with/without high risk of bias; FEM/REM.

Trial sequential analysis (TSA) was performed if there were more than 7 included studies in the meta-analysis. We applied TSA version 0.9.5.10 (Copenhagen: The Copenhagen Trial Unit, Center for Clinical Intervention Research, 2017) to calculate the required sample size in a meta-analysis and to detect the robustness of the result. We used the diversity-adjusted required information size estimated from a control event proportion of the included trials and a priori intervention effect of 5%, and the diversity which was estimated in the included trials.

The GRADE (Grading of Recommendations Assessment, Development and Evaluation criteria) was conducted to assess the quality of evidence for each primary outcome (with synthesized results). Factors that downgraded the quality include imprecision, inconsistency, indirectness, limitations, and bias of the evidence.

## 4 Results

### 4.1 Description of the studies

**Figure 1:**
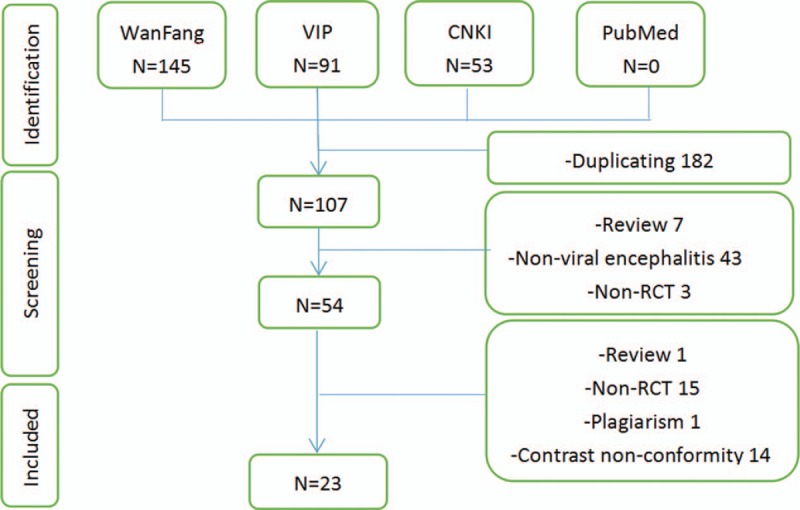
Study flowchart.

After searching the predefined 4 databases, we got 289 citations. Through removing the duplicated literatures among databases and those obviously did not meet the criteria by reading the title and abstract, 54 full text of the papers were downloaded for the further screening. Finally, 23 trials^[10–32]^ were included this review, details of the literature screening flow chart were shown in Figure 1.

All trials were conducted and published in China from 2002 to 2016. They all declared to be randomized controlled trials with 2 parallel groups which compared combination of XNJ injection and other treatments to other treatments alone. According to the age of the participants, 15 trials concerned the VE patients whose age were under 14 years old, and the other 8 trials included patients whose age were over 18 years old. For those who were still children, XNJ injection was mainly given as 0.4–0.6 mL/kg per day once daily, which depended on the weight of the participants; and for the elder patients (whose age were over 18 years old), the XNJ injection was mainly given as 20 mL once daily. The basic treatment that was equally in 2 groups was the conventional therapy of this disease. It may include antibiotics, antiviral drugs, intracranial decompression, vitamin supplement, and maintain of electrolyte balance. According to whether antiviral drugs used as conventional therapy, we classified the included studies in 2 subgroups. Actually only 4 trials^[10,11,14,18]^ did not employ antiviral drugs, in the other 19 trials antiviral drugs (such as acyclovir or ganciclovir) were used in both groups.

Totally 1757 patients were included in this review, with average 38 patients in each group. Proportion of the female patients was almost half of the participants (46.18%). For the patients whose age under 14 years old, the average age of them was between 4.8 and 7.3 years old; and for the adults their average age was between 35.6 and 56.7 years old.

The primary outcome was reported in 19 included trials, in which only 8 trials reported the mortality rate. Details of the characteristics of included trials were shown in Table 1.

**Table 1:**
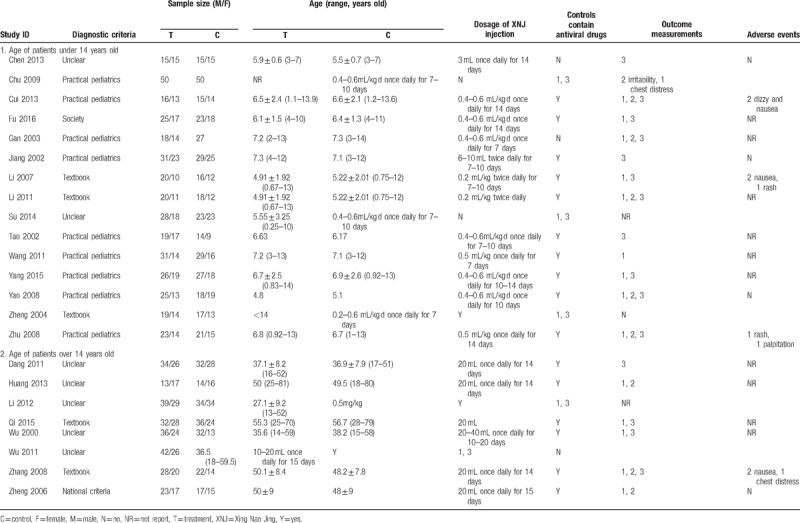
Characteristics of the 23 included trials.

### 4.2 Risk of bias in included studies

According to the criteria we mentioned above, 21 included trials were assessed as having unclear risk of selection bias, since only other 2 included trials^[11,24]^ reported random number table was used for randomization. However, allocation concealment was not reported in any of them.

None of the trials reported the information of blinding methods, since no trial employed placebo control we believed that blinding to participants was impossible to be used. Considering majority of the patients were in a state of coma, the absence of blinding methods may not have serious impact for some of the outcome measurement. Methods of blinding to outcome assessors were also unclear with insufficient information, thus, all trials were assessed as unclear risk of bias on the 2 items of blinding.

**Figure 2:**
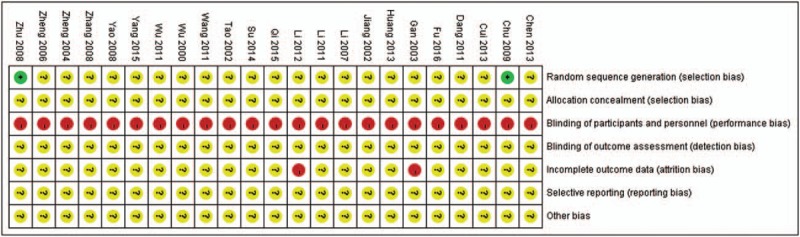
Summary of assessment of risk of bias of 23 included trials.

Two trials^[14,27]^ had obvious imbalance drop-out rate between groups, no appropriate statistical method was used to handle the missing data. So, these 2 trials were evaluated as having high risk of attribution bias. All of the remaining 21 included trials were evaluated as having unclear risk of attribution bias, reporting bias and other bias due to the insufficient information for judgement. Details of the results of risk of bias assessment were shown in Figure 2 and Table 2  .

**Table 2:**
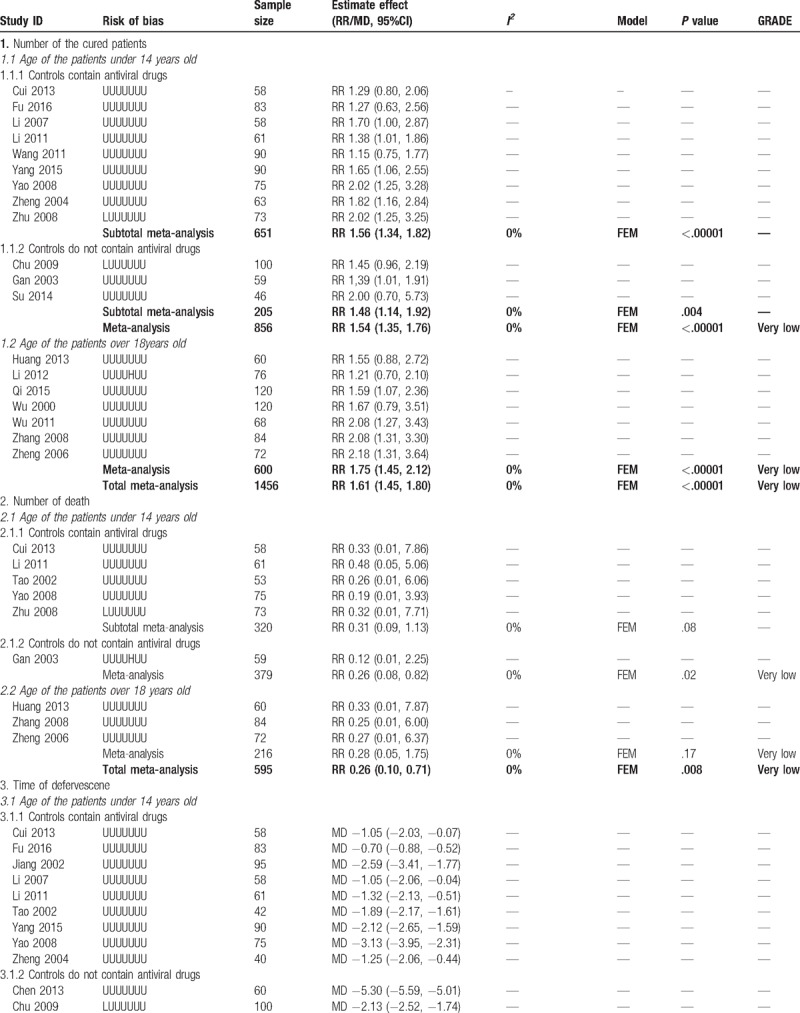
Combined and individual results from the included trials.

**Table 2 (Continued):**
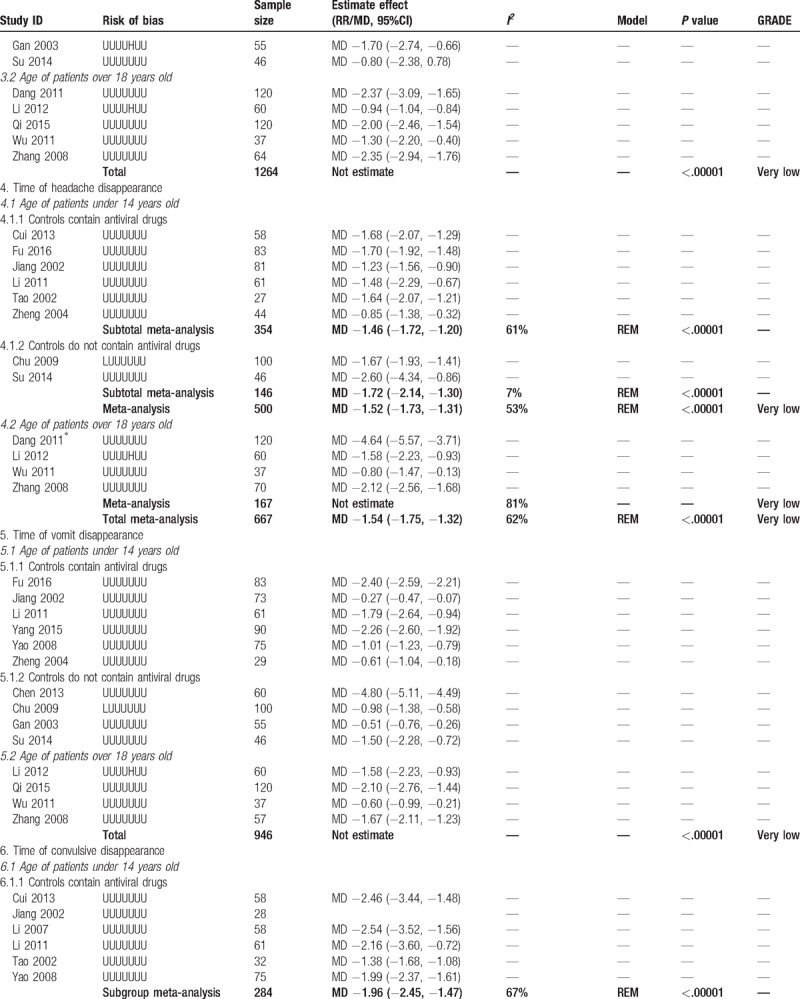
Combined and individual results from the included trials.

**Table 2 (Continued):**
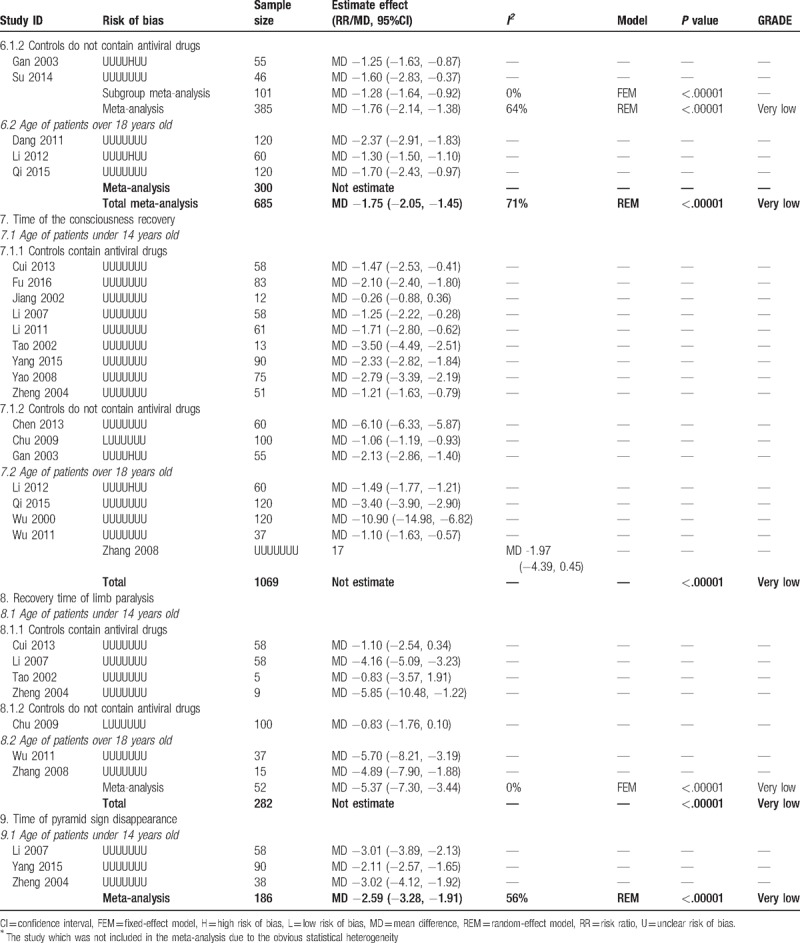
Combined and individual results from the included trials.

### 4.3 Effects of intervention

According to the age of the patients and whether antiviral drug was used in control group, we conducted subgroup meta-analysis to assess the add-on effect of XNJ injection on relevant outcomes. All the trials with patients whose age were over 18 years old used antiviral drugs, so only trials with children could be included in subgroup analysis when classified trials according to the types of conventional therapy. Details of the results from each individual study and the meta-analysis were shown in Table 2  .

#### 4.3.1 Primary outcome: number of the cured patients

**Figure 3:**
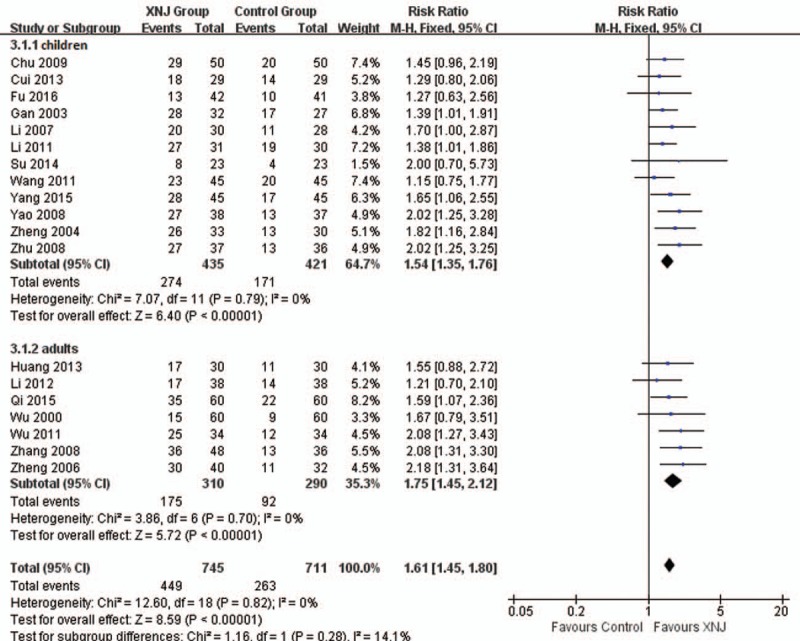
Forest plot of comparison between XNJ plus others and others for outcome: Number of the cured patients. XNJ = Xing Nao Jing.

Nineteen trials reported the numbers of the cured patients after treatment. The results showed combined with XNJ injection may help on increasing the number of cured patients (RR 1.61, 95%CI 1.45 to 1.80, *I*^*2*^ = 0%, *P < *.00001, 19 trials, 1456 participants, Fig. 3), both for children (RR 1.54, 95%CI 1.35–1.76, *I*^*2*^ = 0%, 12 trials, 856 participants) and adults (RR 1.75, 95%CI 1.45–2.12, *I*^*2*^ = 0%, 7 trials, 600 participants). Subgroup analysis also found consistent results no matter antiviral drugs used in control group (RR 1.56, 95%CI 1.34–1.82, *I*^*2*^ = 0%, 9 trials, 651 participants) or not (RR 1.48, 95%CI 1.14–1.92, *I*^*2*^ = 0%, 3 trials, 205 participants).

#### 4.3.2 Primary outcome: Number of death

**Figure 4:**
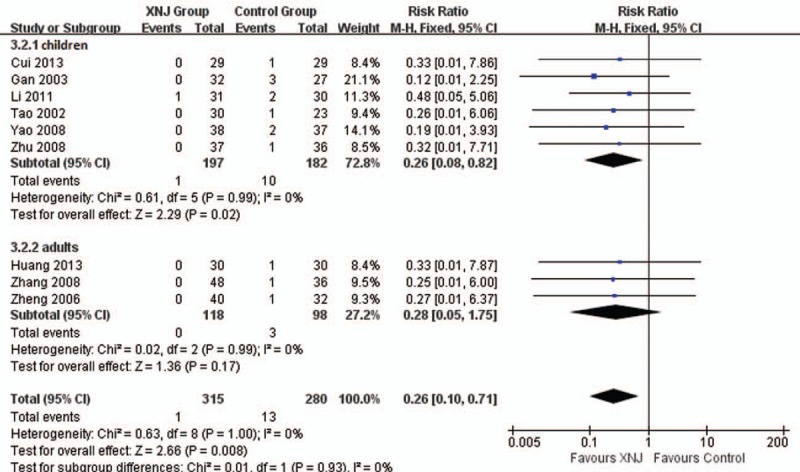
Forest plot of comparison between XNJ plus others and others for outcome: Number of the death. XNJ = Xing Nao Jing.

Nine trials reported the numbers of death. Meta-analysis found better effect on decreasing numbers of deaths in combined group (RR 0.26, 95%CI 0.10–0.71, *I*^*2*^ = 0%, *P = *.008, 9 trials, 595 participants, Fig. 4), and the results were consistent in different types of participants.

#### 4.3.3 Secondary outcomes: the symptoms disappearance time

**Figure 5:**
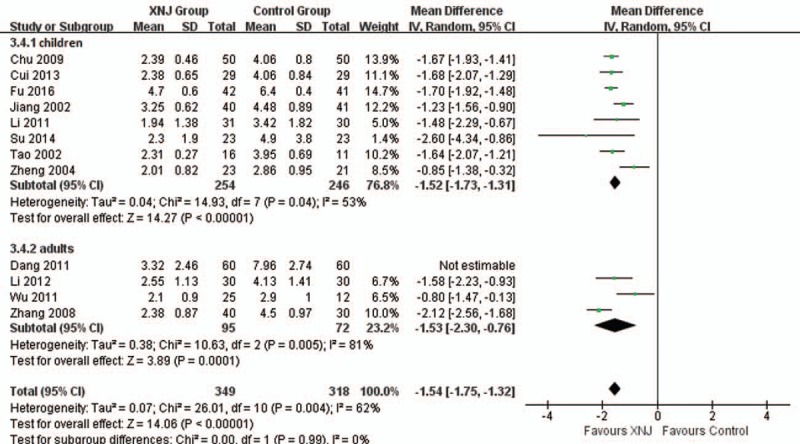
Forest plot of comparison between XNJ plus others and others for outcome: Time of headache disappearance. XNJ = Xing Nao Jing.

Twelve trials reported the time of headache disappearance. Subgroup-analysis showed better adjunctive effect of XNJ injection on decreasing the time of headache (MD −1.52 hours, 95%CI −1.73 to −1.31 hours, *I*^*2*^ = 53%, *P < *.00001, 8 trials, 500 participants) for patients whose age under 14 years old. Overall meta-analysis also showed combination therapy may reduce average 1.54 hours of headache time (MD −1.54 hours, 95%CI −1.75 to −1.32 hours, *I*^*2*^ = 62%, *P < *.00001, 11 trials, 667 participants, Fig. 5) for both children and adults.

Eleven trials reported the time of convulsive disappearance. Overall meta-analysis found better add-on effect of XNJ injection on this outcome (MD −1.75 hours, 95%CI −2.05 to −1.45 hours, *I*^*2*^ = 71%, *P < *.00001, 11 trials, 685 participants), and the subgroup analysis with younger patients had similar results.

Two trials found combined with XNJ injection may reduce the time of recovery of limb paralysis in adults’ patients (MD −5.37 hours, 95%CI −7.30 to −3.44 hours, *I*^*2*^ = 0%, *P < *.00001, 2 trials, 52 participants). Three trials found time of pyramid sign disappearance was shorter in combination therapy group (MD −2.59 hours, 95%CI −3.28 to −1.91 hours, *I*^*2*^ = 56%, *P < *.00001, 3 trials, 186 participants).

For time of defervesce, time of vomit disappearance, time of consciousness recovery, and recovery time of younger patients’ limb paralysis, meta-analysis could not be conducted due to the obvious statistical heterogeneity. However, almost all of them showed significant difference between groups on shortening the time of above symptoms, range of the decreased time was from 0.70 to 5.30 hours for fever, from 1.06 to 10.90 hours for consciousness, from 0.83 to 5.85 hours for limb paralysis of patients whose age under 14 years old. Detail results from individual studies were shown in Table 2   as we mentioned above.

#### 4.3.4 Funnel plot

**Figure 6:**
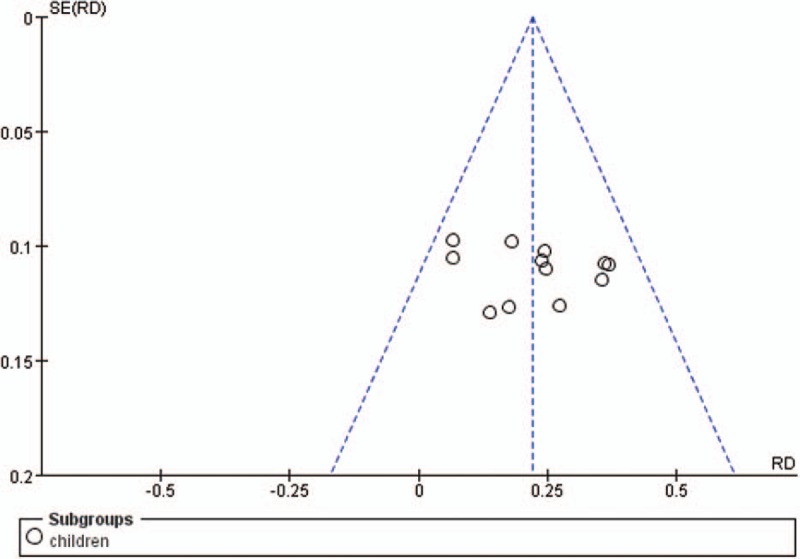
Funnel plot of comparison between XNJ plus others vs. others on the primary outcome: Number of the cured patients in children. XNJ = Xing Nao Jing.

According to the funnel plot of comparison between groups for the primary outcome, we found the potential asymmetry (see Fig. 6) which indicated the possibility of publication bias within the 12 included trials. The figure did not show an inverted funnel shape, probably because the sample sizes of these included studies are similar, and the number of the included studies is limited. Therefore, besides the publication bias, we could not rule out the possibility that the effect of small sample study leads to the asymmetry.

#### 4.3.5 Adverse events

Eleven trials reported the results of adverse events during and after the treatment. Six of them found no adverse event in both groups, and the other 5 trials reported few cases of adverse event in XNJ group (including nausea, rash, palpitation, chest distress, dizzy and irritability), the incidence rate of all kinds of adverse events was less than 7% (2/30) in trials. Due to the insufficient data, difference of the incidence rate of adverse events between groups could not be analyzed.

**Figure 7:**
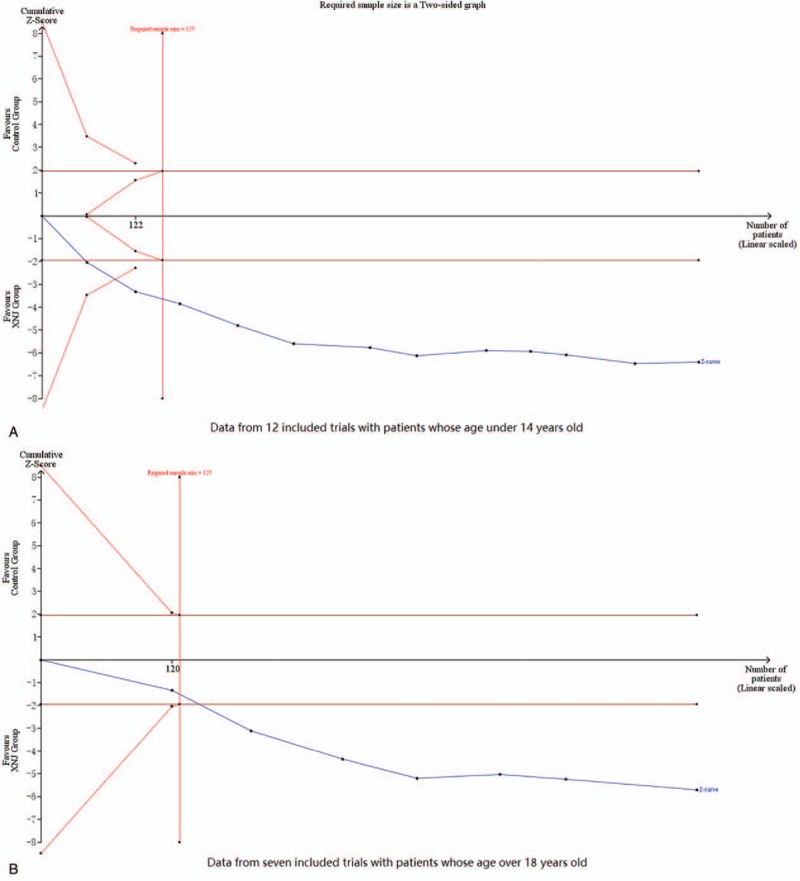
Trial sequential analysis results on increasing numbers of cured patients.

*Trial sequential analysis (TSA)*: We conducted TSA with the data from 2 meta-analyses in which more than 7 trials were included. One was conducted with the data from 12 trials that compared XNJ injection combined conventional therapy to conventional therapy alone on numbers of cured patients whose age under 14 years old. TSA illustrated that the cumulative *Z*-curve across the traditional boundary of 5% significance (horizontal line) and the monitoring boundaries (inward sloping curves) (see Fig. 7A). After the fourth study, the significance testing had been performed each time a new trial was added to the meta-analysis, which meant the sample size achieved the required 157 participants and we had enough power to confirm the evidence (that with adjunction of XNJ injection, the conventional therapy may increase 22% more cured children with VE) controlling for the risk of random error. Similar result was shown in another TSA with data from 7 trials which also compared combination group with conventional therapy alone on numbers of cured patients whose age over 18 years old. TSA also illustrated that the cumulative *Z*-curve across the horizontal line and the inward sloping curves (see Fig. 7B), which meant the sample size achieved the required 127 participants and we had enough power to confirm the evidence (that the combination therapy may increase 24% more cured adults with VE).

## 5 Discussion

### 5.1 Summary of main results

This review involved 23 trials and 1757 participants, results from 5 meta-analyses, 13 subgroup meta-analyses, and the single studies showed that on the basis of conventional therapy XNJ injection (0.4–0.6 mL/kg daily for children, 20 mL/day for adults) may have better effect on increasing the numbers of cured patients and decreasing the time of recovery of main symptoms for patients with viral encephalitis. Patients used combination of XNJ injection and conventional therapy had higher cured rate (average 1.60 times than control) and less mortality rate (average 0.26 times than control), the former was supported by the TSA results. The average difference of time for fever, conscious, or convulsive recovery was average 2hrs shorter in combination group than in control. Safety of the XNJ injection was failed to evaluate due to the insufficient evidence in this review.

### 5.2 Quality of the evidence

Due to the unclear/high risk of bias of all the included trials, the obvious statistical heterogeneity among trials and the potential publication bias, level of the evidence for effect of XNJ injection combined with conventional therapy versus conventional therapy alone for VE were all assessed as “very low” according to the GRADE assessment criteria (see Table 2  , and Table 3 presented the GRADE assessment results for all the primary outcomes). This limited the power to confirm the adjunctive effectiveness of XNJ injection for this condition, future high quality randomized controlled trials are still needed to improve the quality of the evidence.

**Table 3:**
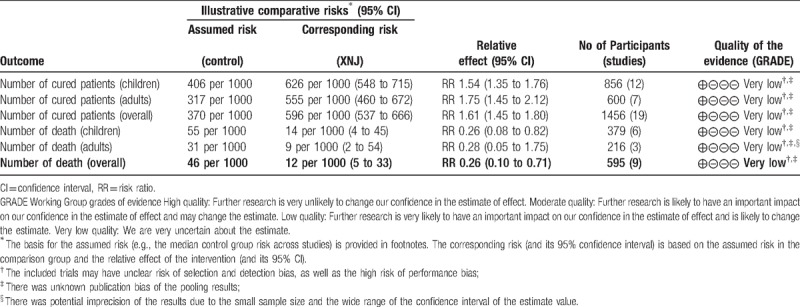
Summary of finding table of XNJ injection in adjunctive to conventional therapy for viral encephalitis.

Compared to the previous review^[8]^ we mentioned above, this review included 9 more trials, and reported more outcome measurements (including mortality rate). Also, we conducted subgroup analysis to compare the difference between children and adults. Though the quality of the evidence was still low, the precision of the estimate effect value in this review was greater than the previous one (with narrower range of confidence interval).

### 5.3 Implications for practice

According to the results, under the assistance of XNJ injection there would be 200 more cured people per thousand patients than conventional therapy single used. At the mean time, the combination of XNJ injection and conventional therapy may save almost 30 more patients from death in each 1000 patients been treated. However, if we count the number needed to treat/harm (NNT/NNH) for this outcome, we got the absolute reduction of risk between groups were 0.23 (95%CI 0.18–0.28) for cure and −0.04 (95%CI −0.07 to −0.01) for death, which means to get one patient cured, 4 patients needed to be treated; and to save one patient from death, 25 patients needed to be treated with XNJ injection and conventional therapy. It seems the combination therapy is of great value in increasing cure rate.

Subgroup analysis did not find significant difference results between children and adults for all the concerned outcomes. However, the youngest patient involved in this review was 5 year old; thus, the results of this review could not be explained beyond this age range. When the control treatments contained antiviral drugs, the combination group seems less superior according to the subgroup analysis (see Table 2  ), but this may be caused by the small study effect or publication bias.

Besides the effectiveness of XNJ injection, we also concerned the safety of this herbal product. There are many reports (e.g., Tarantino et al^[33]^) of adverse reactions suggesting potential safety hazards of herbal medicine, especially its hepatotoxicity, which has aroused international attention. According to the Guiding Principles for Clinical Evaluation of Drug-induced Liver Injury in Traditional Chinese Medicine,^[34]^ which is issued by the State Drug Administration, the clinical diagnosis of herb-induced liver injury should be based on careful understanding of the medical history (especially the medication history), physical examination, etiological examination, immunological examination, genetic examination, biochemical examination, and imaging examination, so as to differentiate the liver diseases caused by other causes. We retrieved 2 research reports^[35,36]^ on postmarketing reappraisal of XNJ injection. Through follow-up observation of nearly 2000 hospitalized patients treated with XNJ injection, no adverse reactions were found. In this review, the included trials reported only 7% patients occurred adverse events, none of them could be defined as liver injury. Thus, although the safety of XNJ injection in the treatment of VE remains to be further verified, there was no evidence to show its potential hepatotoxicity.

Overall, though the level of the evidence is “very low”, we’d love to recommend the application of XNJ injection in addition to the conventional therapy for patients with viral encephalitis, since the significant better estimate effect than conventional treatment alone used. Considering the weak evidence for this intervention based on current clinical studies, the practitioners need to combine their own experience with the actual situation of patients when using the XNJ injection.

### 5.4 Implications for the future researches

Since the advantages regarding add-on effectiveness of XNJ injection were not certain for VE, the cost-effectiveness assessment should be done in the future to determine whether the advantages of combination therapy were still existing in consideration of the economic outcomes.

Besides the effectiveness, safety issue is also concerned for herbal medicine injection. However, few of the published articles mentioned the safety outcomes of this kind of intervention. In this review, only 5 of the included trial reported the adverse events during treatment, thus, no conclusion could be drawn for the safety of XNJ injection. We suggest that future researches should report safety outcomes relevant to the treatment of XNJ injection.

Furthermore, the low methodological quality of the included RCTs limited the level of the evidence, future studies should also aware the potential bias during the research and try to improve the quality of the trials.

## 6 Conclusions

This review found the potential effectiveness of combination of XNJ injection and conventional therapies for VE, especially on increasing the number of cured patients. Due to the poor methodological quality of the included studies, the level of the evidence could only be defined as “very low” according to the GRADE criteria. More high-quality trials are still needed to prove the superior effect of XNJ injection as adjunctive treatment for this disease. Safety issue is also concerned, and conclusion could only be drawn on the effectiveness of the XNJ injection as add-on treatment for VE patients on increasing the cured rate according to the TSA results. Firm conclusion on other outcome measures for effectiveness assessment or safety of XNJ injection could not be draw according to this review due to the insufficient evidence.

## Author contributions

**Conceptualization:** Jiarui Wu.

**Data curation:** Huijuan Cao, Shibing Liang, Wei Zhou.

**Formal analysis:** Huijuan Cao.

**Funding acquisition:** Huijuan Cao, Jiarui Wu.

**Methodology:** Huijuan Cao.

**Project administration:** Jiarui Wu.

**Supervision:** Jiarui Wu, Chengliang Zhang.

**Validation:** Huijuan Cao, Wei Zhou, Jiarui Wu, Chengliang Zhang.

**Writing – original draft:** Huijuan Cao.

**Writing – review & editing:** Huijuan Cao, Shibing Liang, Wei Zhou, Jiarui Wu, Chengliang Zhang.
